# Stable expression of promyelocytic leukaemia (PML) protein in telomerase positive MCF7 cells results in alternative lengthening of telomeres phenotype

**DOI:** 10.1186/2041-9414-3-5

**Published:** 2012-08-27

**Authors:** Jacklyn W Y Yong, Xiujun Yeo, Md Matiullah Khan, Martin B Lee, M Prakash Hande

**Affiliations:** 1 Department of Physiology, Yong Loo Lin School of Medicine, National University of Singapore, Singapore, 117597, Singapore; 2 Cancer Science Institute, National University of Singapore, Singapore, 117456, Singapore; 3 Tembusu College, National University of Singapore, Singapore, 138598, Singapore; 4 Department of Physiology, Yong Loo Lin School of Medicine, National University of Singapore, MD6, #14-02P, 14 Medical Drive, Singapore, 117599, Singapore

## Abstract

**Background:**

Cancer cells can employ telomerase or the alternative lengthening of telomeres (ALT) pathway for telomere maintenance. Cancer cells that use the ALT pathway exhibit distinct phenotypes such as heterogeneous telomeres and specialised Promyelocytic leukaemia (PML) nuclear foci called APBs. In our study, we used wild-type PML and a PML mutant, in which the coiled-coil domain is deleted (PML C/C^-^), to investigate how these proteins can affect telomere maintenance pathways in cancer cells that use either the telomerase or ALT pathway.

**Results:**

Stable over-expression of both types of PML does not affect the telomere maintenance in the ALT cells. We report novel observations in PML over-expressed telomerase-positive MCF7 cells: 1) APBs are detected in telomerase-positive MCF7 cells following over-expression of wild-type PML and 2) rapid telomere elongation is observed in MCF7 cells that stably express either wild-type PML or PML C/C^-^. We also show that the telomerase activity in MCF7 cells can be affected depending on the type of PML protein over-expressed.

**Conclusion:**

Our data suggests that APBs might not be essential for the ALT pathway as MCF7 cells that do not contain APBs exhibit long telomeres. We propose that wild-type PML can either definitively dominate over telomerase or enhance the activity of telomerase, and PML C/C^-^ can allow for the co-existence of both telomerase and ALT pathways. Our findings add another dimension in the study of telomere maintenance as the expression of PML alone (wild-type or otherwise) is able to change the dynamics of the telomerase pathway.

## Background

Telomeres are specialised chromatin structures capping the ends of chromosomes [1]. They consist of TTAGGG repeats and function to protect the ends of the DNA. Human telomeres in somatic cells lose about 50 to 150 base pairs with each round of cellular division. When telomeres shorten to a critical length, cells will enter a state of permanent growth arrest termed as replicative senescence. Cellular senescence limits the proliferative capacity of cells and this suppresses tumourigenesis [2]. To overcome cellular senescence and to achieve immortality, cancer cells maintain telomeres through the activation of the telomerase enzyme. Some cancer cells also use the lesser-known telomere maintenance pathway-the Alternative Lengthening of Telomeres pathway, ALT [3,4].

Cancer cells that employ the ALT pathway for telomere maintenance exhibit distinct hallmarks from cells that use telomerase [5]. These cells exhibit heterogeneous telomeres, with length varying between 3 kb and 50 kb [5-7]. ALT cancer cells also contain ALT-associated promyelocytic leukaemia (PML) bodies, termed APBs [5]. While APBs contain normal constituents of PML bodies such as PML and Sp100 proteins, they also contain telomeric DNA and telomere-associated proteins such as telomere-repeat binding factors (TRF) 1 and 2 [8]. APBs are unique to ALT cells as they are not present in normal and telomerase-positive cells [9]. As APBs and heterogeneous telomere length are exclusive to cells using the ALT pathway for telomere maintenance, these two phenotypes are used as definitive hallmarks for ALT cells [10].

There exist uncertainties as to whether APBs are truly involved in the ALT pathway or if they are simply by-products of the pathway [11]. APBs have been proposed to be the sites of telomeric recombination by virtue of the detection of telomeric DNA, and DNA repair and recombination proteins within them [8,12]. This suggests APBs as important sites for the co-localisation of proteins involved in ALT and reinforce the hypothesis that telomeric recombination is the mechanism of telomere elongation in the ALT pathway [5,13,14]. However, the absence of APBs in some cells that possess telomerase-independent telomere maintenance mechanisms illustrates that APBs might not be absolutely essential for the ALT pathway [15,16]. Still, APBs are believed to be tightly correlated to the ALT pathway as important mediators for the process, as studies have shown the appearance and disappearance of APBs with the onset and inhibition of ALT pathway, where the inhibition of APBs caused telomere shortening [8,17,18].

The PML protein is a main component of PML nuclear bodies. There are three small ubiquitin-like modifier (SUMO) binding sites in PML, at lysines 65, 160 and 490 [19]. As SUMOylation of PML has been shown to affect the formation of PML nuclear bodies [20], such modification is deemed to be important for the formation of PML nuclear bodies [19-21]. However, a SUMO interaction motif present in PML, [22] allows it to tether to SUMO independently of the three SUMO lysine sites. It is thus likely that the SUMO-modification of PML is also important for APBs formation, although, to the best of our knowledge, the importance of the SUMOylation of PML in the formation of APBs and thereby in the ALT pathway has not been studied. The PML protein contains a coiled-coil domain, which has been shown to be important in the initiation of the nucleation and oligomerisation processes that are critical in the formation of PML nuclear bodies [21,23,24]. This domain is also required for the critical step in the SUMOylation process of binding of Ubc9, a SUMO conjugation enzyme, to PML [24]. Thus, the coiled-coil domain of PML is probably also important for the formation of APBs. However, it is unclear if the coiled-coil domain can affect the ALT pathway in part due to its influence on APBs formation.

In our study, we investigated if PML affects the formation of APBs through SUMOylation, the importance of APBs in the ALT pathway, and if PML has any impact on the telomerase pathway. We expressed SUMO-defective and coiled-coil domain deficient PML in ALT cells to determine if these exogenous proteins can form APBs. Over-expression of both wild-type and mutant PML was also performed in telomerase-positive MCF7 cells to study if they affect telomere maintenance regulated by telomerase. Here, we present our novel finding of the detection of APBs in telomerase-positive MCF7 cells following stable expression of wild-type PML. Our study also showed for the first time that while the expression of wild-type and mutant PML did not affect the telomere phenotype of the ALT cells, they brought about a dramatic elongation of the telomeres in the telomerase-positive cells. Interestingly, such elongation could occur in the absence of APBs. Elongation of telomeres in MCF7 cells was accompanied with changes in telomerase activity and this is apparently dependent on the type of PML over-expressed. Our study thus suggests that while APBs might definitively promote the onset of the ALT pathway, telomere elongation may proceed in the absence of APBs.

## Results

**Figure 1:**
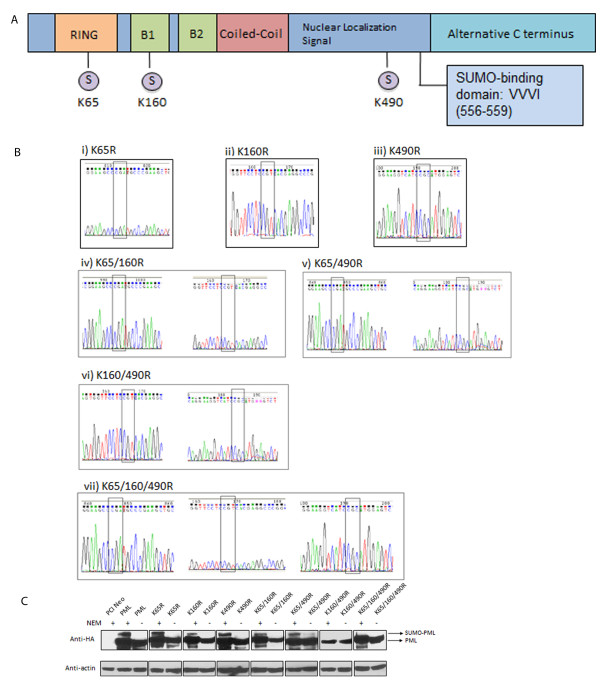
**A) Schematics of the PML protein depicting its functional domain.** RING indicates the RING finger domain while B1 and B2 represent the B-boxes. S indicates SUMO protein modification of the lysine residues at the indicated positions (boxed in red). **B**) Chromatographs depicting successful mutations of the lysine sites to arginine in the PML protein through PCR mutagenesis. Chromatograms of (i) PML K65R, (ii) K160R, (iii) K490R, (iv) K65/160R, (v) K65/490R, (vi) K160/490R and (vii) K65/160/490R plasmids. The boxed codons indicate the location of the successful mutations. **C**) Western blot analysis showed that PML K160/490R double and K65/160/490R triple mutants are not modified by SUMO. JFCF-6/T.1R cells were transfected with the indicated plasmids and cells were harvested 48 h after transfection in the presence and absence of NEM. The use of NEM preserves SUMO-conjugated proteins. The SUMO protein adds about 10–20 KDa in molecular weight to that of the protein in its native form. The slower migrating band is indicative of SUMO-modified PML. All PML constructs are HA-tagged and the blots were probed with the indicated antibodies (anti-HA: 1:5000, anti-actin: 1:2000). Actin was used as a loading control.

To determine the effects of SUMO-defective PML on the formation of APBs, we first generated PML KR plasmids where the SUMO lysine sites were mutated to arginine. There are three SUMO attachment sites in PML [19], at lysine 65, 160 and 490 (Figure 1A). A total of seven mutants were generated (Figure 1B); three single mutants containing mutation at each lysine site (Figure 1B i-iii), three double mutants containing mutation at two lysine sites (Figure 1B iv-vi) and one triple mutant containing mutations at all the three sites (Figure 1B vii). These mutants are hence termed PML KR mutants. All seven mutants, in addition to the wild-type PML protein, are tagged with haemagglutinin (HA), to allow for the detection of exogenous PML proteins. N-ethylmaleimide (NEM) is commonly used to preserved SUMO-conjugated proteins [25], allowing for their detection in western blots. Thus protein lysates were harvested in the presence and absence of NEM. As SUMO add about 20 kDa in molecular weight, SUMO-modified PML appears as the slower migrating band. All PML KR mutants remained modifiable by SUMO except for the K160/490R double mutant and the triple mutant (Figure 1C).

**Figure 2:**
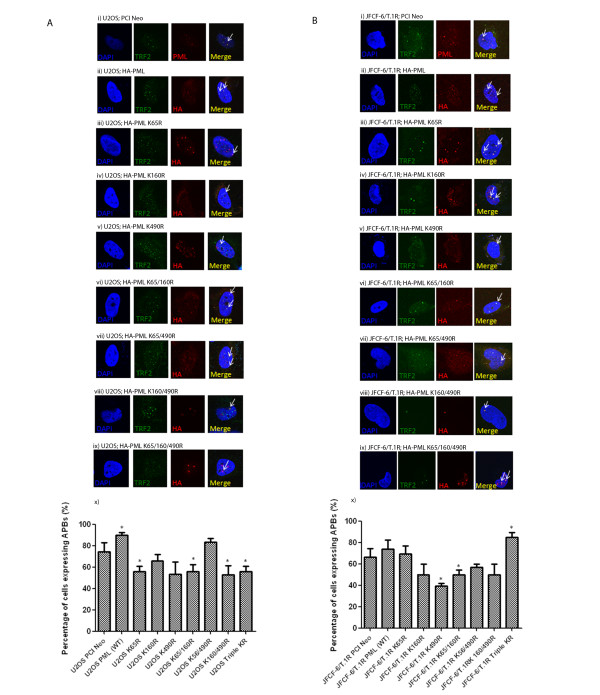
**The formation of APBs is independent of the SUMOylation of the PML protein.** All PML KR mutants can form APBs in ALT cells. **A**) U2OS and **B**) JFCF-6/T.1R cells were transfected with (i) empty vector PCI Neo, (ii) wild-type PML, (iii) PML K65R, (iv) PML K160R, (v) PML K490R, (vi) PML K65/160R, (vii) PML K65/490R, (viii) PML K160/490R and (ix) PML triple K65/160/490R plasmids. (x) Graphs depicting percentage of cells expressing APBs. At least 50 cells were scored and the results were summarised from at least three independent experiments. Values represent the mean ± SD. All PML plasmids are HA-tagged. In PCI Neo transfected cells (i), detection of APBs was based on co-localisation of endogenous PML and TRF2. Arrows indicate detection of APBs, defined as the co-localisation of PML with TRF2. Immunofluorescence was performed 48 h after transfection and the indicated antibodies were used. DAPI was used for nuclear staining. Images were captured with confocal microscopy at 100 X magnification. * indicates p < 0.05.

We then investigated the effects of PML KR mutants on the formation of APBs in ALT cells. In our study, APBs are defined by the co-localisation of PML with TRF2. As the PML constructs used in this study are HA-tagged, anti-HA and anti-TRF2 antibodies were used for co-immunofluorescence. The use of anti-HA antibodies eliminates confusion between endogenous and exogenously introduced PML. The ALT-positive U2OS and JFCF-6/T.1R cells were transiently transfected with wild-type PML and PML KR mutants (Figure 2). Co-localisation of PML and TRF2 was detected with all PML KR mutants, suggesting that the formation of APBs is independent on the SUMOylation of the PML protein.

**Figure 3:**
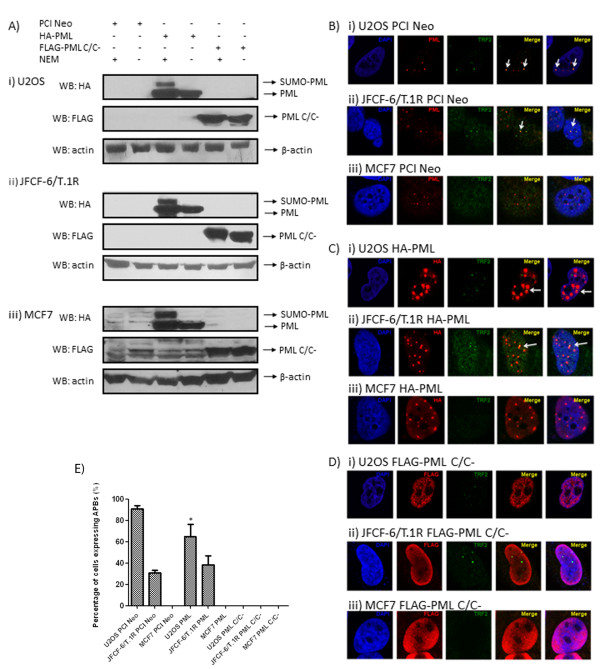
**A) PML C/C**^**-**^**is not modified by SUMO in (i) U2OS, (ii) JFCF-6/T.1R and (iii) MCF7 cells.** U2OS and JFCF-6/T.1R are established ALT cell types while MCF7 is a telomerase-positive cell line. The cells were transfected with the indicated plasmids. Cell lysates were harvested in the presence and absence of N-ethylmaleimide (NEM) 48 h after transfection and protein expression was detected with western blot analysis. Wild-type PML is HA-tagged while PML C/C^-^ is FLAG-tagged. Blots were probed with the indicated antibodies. B-actin was used as a loading control. **B**) APBs are ALT-specific. Endogenous APBs were detected in (i) U2OS and (ii) JFCF-6/T.1R cells but not in (iii) MCF7 cells. PML antibodies were used to detect endogenous PML. ( **C**) Transiently over-expressed HA-tagged wild-type PML co-localized with TRF2 in (i) U2OS and (ii) JFCF-6/T.1R but not in non-ALT (iii) MCF7 cells. ( **D**) Transiently over-expressed FLAG-tagged coiled-coil domain deficient PML mutant did not form distinct nuclear bodies in all three cell-lines, (i) U2OS, (ii) JFCF-6/T.1R, (iii) MCF7. **E**) Graph depicting the percentage of cells expressing APBs. At least 100 cells were scored and the results were summarized from at least three independent experiments. Values represent the mean ± SD. APBs are defined by the co-localisation of PML with TRF2 and are indicated by arrows. PCI Neo was used as an empty vector control. DAPI was used for visualisation of the nucleus and the indicated antibodies were used. Images were captured with confocal microscopy at 100 X magnification. * indicates p < 0.05.

The PML protein contains a coiled-coil domain which is critical for the nucleation process in the formation of nuclear bodies [21]. Interestingly, the coiled-coil domain is also implicated in the SUMOylation of PML whereby the loss of the domain leads to a loss of SUMOylation of the PML-retinoic acid receptor alpha fusion protein [24]. We first verified that the PML protein lacking the coiled-coil domain (henceforth referred to as PML C/C^-^) does not exhibit a higher molecular weight band indicative of SUMO-PML (Figure 3A) in three different cell lines. We then questioned whether the coiled-coil domain might affect the formation of APBs in ALT cells. To this end, we performed transient transfections of wild-type PML and PML C/C^-^ plasmids into U2OS, JFCF-6/T.1R and MCF7 cells to carry out immunofluorescence studies (Figure 3B-D). We observed that the over-expressed PML in U2OS and JFCF-6/T.1R ALT cells co-localised with TRF2, giving rise to APBs. However, the over-expressed PML in MCF7 cells did not form APBs even though distinct PML foci were formed. It should be noted that while TRF2 foci were easily observed in ALT cells, they were not readily detected in MCF7 cells. Interestingly, over-expression of PML C/C^-^ in all the three cell types did not form any PML distinct foci that allowed for the formation and observation of APBs.

**Figure 4:**
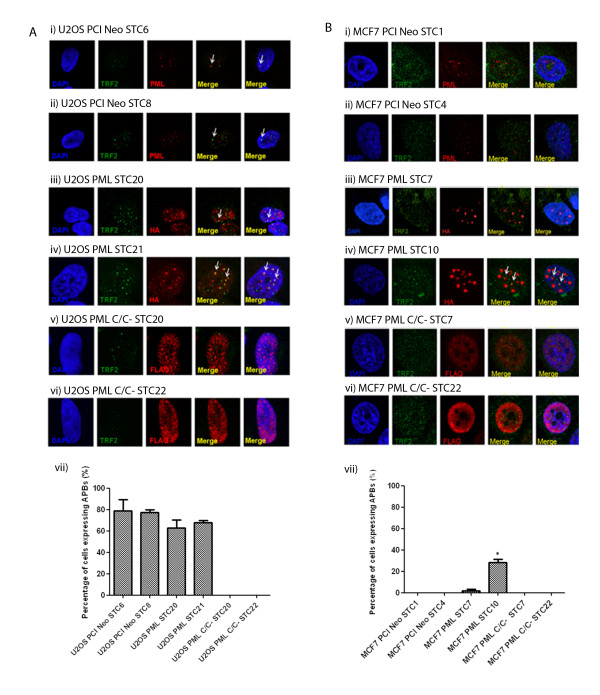
**Immunofluorescence studies of stably-expressed wild-type PML and PML C/C**^**-**^**in U2OS and MCF7 cells.** ( **A**) Endogenous APBs were detected in (i) U2OS PCI Neo STC6 and (ii) PCI Neo STC8 clones. APBs were detected in (iii) U2OS PML STC20 and (iv) PML STC21 clones which stably express wild-type PML. APBs were not formed with the stable expression of PML C/C^-^ in (v) U2OS PML C/C^-^ STC20 and (vi) PML C/C^-^ STC22 clones. (vii) Graph depicting percentage of U2OS cells expressing APBs. (**B**) Novel detection of APBs in MCF7 cells stably expressing wild-type PML. APBs were not present in (i) MCF7 PCI Neo STC1 and (ii) STC4 clones. No APBs were detected in (iii) MCF7 PML STC7 clone but stably-expressed wild-type PML formed distinct foci that co-localized with TRF2 to form APBs in (iv) PML STC10 clone. PML C/C^-^ did not form APBs when expressed stably in MCF7 cells, (v) PML C/C^-^ STC7 and (vi) PML C/C^-^ STC22. (vii) Graph depicting percentage of MCF7 cells expressing APBs. At least 100 cells were scored and the results were summarized from at least three independent experiments. Values represent the mean ± SD. Wild-type PML is HA-tagged while PML C/C^-^ is FLAG-tagged. APBs are defined by the co-localisation of PML with TRF2 and are indicated by arrows. DAPI was used for visualisation of the nucleus and the indicated antibodies were used. Images were captured with confocal microscopy at 100 X magnification. * indicates p < 0.05.

In this study, we wanted to investigate how the prevention of APBs formation can affect telomere maintenance through the ALT pathway. As changes in telomere length are more prominent in long-term studies, we generated U2OS and MCF7 cells that express wild-type PML and PML C/C^-^ stably. For each of the empty vector pCI Neo, wild-type PML and PML C/C^-^ constructs, two clones of U2OS and MCF7 cells were randomly selected and used in subsequent studies. Immunofluorescence was performed on the clones to determine if the long-term expression of wild-type PML and PML C/C^-^ affects the formation of APBs. In the two U2OS clones that stably express wild-type PML, U2OS PML STC20 and PML STC21, PML continued to form distinct foci that co-localised with TRF2 to form APBs (Figure 4A). In the two U2OS clones stably expressing PML C/C^-^, U2OS PML C/C^-^ STC20 and PML C/C^-^ STC22, the stable over-expression of PML C/C^-^ did not lead to the formation of distinct foci to result in APBs.

Interestingly, in the two MCF7 clones that stably express wild-type PML, different phenotypes were observed (Figure 4B). In MCF7 PML STC7, while PML formed distinct foci, no APBs were observed. However, in MCF7 PML STC10, the distinct PML foci formed co-localised with TRF2 to give rise to APBs. To the best of our knowledge, this is the first study to show the presence of APBs following the stable expression of wild-type PML in MCF7 cells.

**Figure 5:**
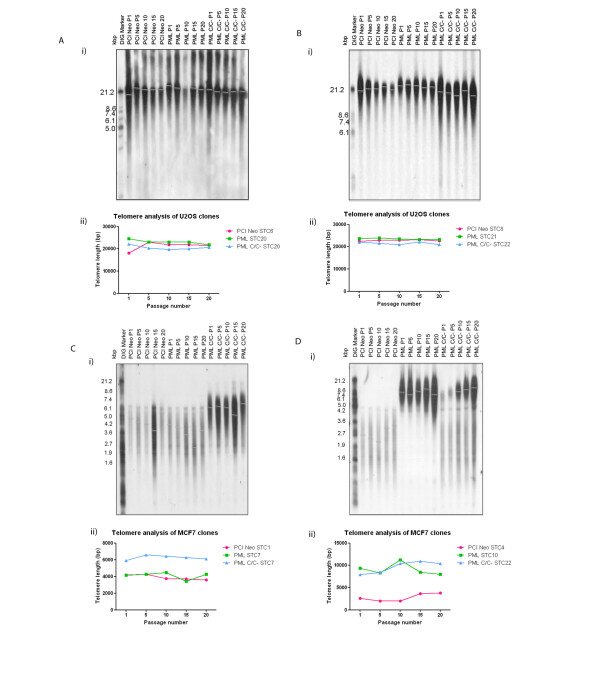
**Terminal restriction fragment analysis coupled with Southern blot was used for telomere length analysis.** The stable over-expression of wild-type PML and PML C/C^-^ did not affect telomere length in U2OS cells. **A**) (i) Southern blot analyses of telomere length in U2OS PCI Neo STC6, PML STC20 and PML C/C^-^ STC20. (ii) Graphical representation of telomere length of clones in (**A**) (i). **B**) (i) Southern blot analyses of telomere length in U2OS PCI Neo STC8, PML STC21 and PML C/C^-^ STC22. (ii) Graphical representation of telomere length of clones in (**B**) (i). Telomere length changes were observed in MCF7 cells following stable expression of wild-type PML and PML C/C^-^. **C**) (i) Southern blot analyses of telomere length in MCF7 PCI Neo STC1, PML STC7 and PML C/C^-^ STC7. (ii) Graphical representation of telomere length of clones in (**C**) (i). **D**) Southern blot analyses of MCF7 PCI Neo STC4, PML STC10 and PML C/C^-^ STC22. (ii) Graphical representation of telomere length of clones in (**D**) (i). Genomic DNA was obtained from the cells at every 5^th^ passage until the 20^th^ passage. Genomic DNA was digested with HinfI and RsaI and the digested DNA was resolved on an agarose gel. Southern blot analysis was carried out using the TeloTAGGG telomere length assay kit according to manufacturer’s protocol (Roche). The smears represent the telomeres detected and grey lines indicate the mean telomere length as analysed with Kodak Imager.

As APBs are thought to be essential for the ALT pathway, we speculated that the absence of APBs in U2OS PML C/C^-^ clones and the presence of APBs in MCF7 cells might affect telomere length in these cells. We then looked at the telomere status in the clones for 20 passages, with telomere length analysis performed at every 5^th^ passage (Figure 5). The mean telomere length was measured through Southern blot analysis of terminal restriction fragments. In both U2OS PML STC20 (Figure 5A) and PML STC21 (Figure 5B) clones, wild-type PML did not positively affect telomere length. Similarly, PML C/C^-^ did not negatively affect telomere length in U2OS PML C/C^-^ STC20 and PML C/C^-^ STC22 clones.

In MCF7 cells, the stable expression of wild-type PML in MCF7 PML STC7 did not affect telomere length (Figure 5C). However, in MCF7 PML STC10, the long-term expression of wild-type PML led to a dramatic increase in telomere length (Figure 5D). Telomere length in MCF7 PML STC10 cells increased from about 3000 base pairs to 8000 base pairs. It is noteworthy that APBs were observed in MCF7 PML STC10 cells as mentioned above (Figure 3B). Interestingly, the stable expression of PML C/C^-^ led to telomere elongation in both MCF7 PML C/C^-^ STC7 and STC22 clones. While the telomere length in MCF7 PML C/C^-^ STC22 increased from 3000 to 9000 base pairs, telomere length doubled in MCF7 PML C/C^-^ STC7. It should also be noted that telomere length phenotypes in MCF7 PML STC10 and PML C/C^-^ STC22 are reminiscent of that in ALT cells. We hereby report, for the first time, that stably expressed wild-type PML and PML C/C^-^ can bring about a rapid increase in telomere length in MCF7 cells.

**Figure 6:**
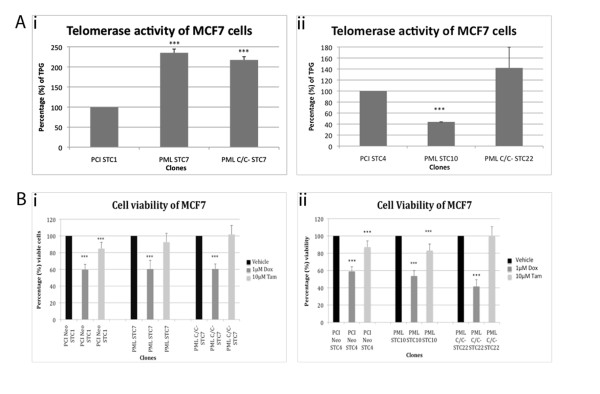
**A) Telomerase activity in MCF7 cells is affected by wild-type PML and PML C/C**^**-**^**.** (i) Telomerase activity in MCF7 PML STC7 and PML C/C^-^ STC7 cells are increased compared to the MCF7 PCI Neo STC1 cells. (ii) Telomerase activity was reduced in MCF7 PML STC10 while PML C/C^-^ STC22 demonstrated increased activity. Telomerase activity was measured using the TRAPezeXL Telomerase Detection kit according to manufacturer’s protocol (Millipore). Telomerase activity is represented in terms of percentage of the total product generated (TPG) as measured by the assay. B) MCF7 clones demonstrating extremely long telomeres (Figure 5D i) exhibited enhanced susceptibility towards doxorubicin. (i) There were no significant differences in the susceptibility of MCF7 PML STC7 and PML C/C^-^ STC7 towards doxorubicin or tamoxifen. (ii) MCF7 PML STC10 and PML C/C^-^ STC22 demonstrated an increased sensitivity toward doxorubicin. Cell viability was measured using the crystal violet assay. Cells were treated with either 1 μM of doxorubicin or 10 μM of tamoxifen for 24 h. DMSO was used as a vehicle control. At least three independent experiments were performed. *** indicates p < 0.01.

We then studied the telomerase activity in the MCF7 clones to determine if there might be any changes to its inherent telomere maintenance mechanism resulting from the over-expression of the PML proteins. Telomerase activity was compared to the cells stably expressing the empty vector, PCI Neo (Figure 6A). In MCF7 PML STC7 cell type where there was no apparent telomere length changes, telomerase activity was more than doubled compared to that in the empty vector control cells. However, in MCF7 PML STC10, wherein there was a rapid increase in telomere length, the telomerase activity was significantly reduced. This suggests a reduction in the role of the telomerase enzyme in maintaining and/or elongating telomeres. Interestingly, telomerase activity increased in MCF7 PML C/C^-^ STC7 and STC22 clones, both of which demonstrated telomere elongation. Collectively, our data suggests that the stable expression of wild-type PML and PML C/C^-^ in telomerase-positive MCF7 cells affects the inherent telomere maintenance mechanism. The changes could result from a switch in the pathway utilised, from a dominant telomerase pathway to the ALT pathway, or to a state of co-existence of the two pathways of telomere maintenance.

As it appears that the pathway of telomere maintenance in MCF7 cells is affected by the stable expression of wild-type PML and PML C/C^-^, we sought to determine the clinical implications of such changes, if any. To this end, we used doxorubicin and tamoxifen, which are being used in the clinics for breast tissue malignancies [26,27], to study the cell viability of the MCF7 clones following 24 h of treatment with 1 μM of doxorubicin or 10 μM of tamoxifen (Figure 6B). In addition to the MCF7 cells being more sensitive to doxorubicin than to tamoxifen, it appears that MCF7 PML STC10 and MCF7 PML C/C^-^ STC22 cells, in which rapid telomere elongation is observed, are slightly more sensitive to doxorubicin. Our results suggest that a change in telomere maintenance mechanism might affect the susceptibility of cancer cells to therapeutic compounds.

## Discussion

As APBs are highly specific to cancer cells utilising the ALT pathway for telomere elongation, they could be potentially useful tools in novel anti-cancer drug discovery. While the exact extent of the requirement and involvement of APBs in the initiation and propagation of the ALT pathway is unclear, APBs are deemed as essential components for the pathway. While the role of APBs has not been studied in detail, it is certain that the PML protein is required for the formation of APBs [20].

In the present study, wild-type PML and a PML mutant, in which the coiled-coil domain (PML C/C^-^) has been deleted, were expressed in ALT and telomerase positive cells to determine if they can affect both types of cells which utilise distinct telomere maintenance mechanisms. The stable over-expression of wild-type PML and PML C/C^-^ in the ALT cell type, U2OS, did not result in any changes in APBs formation and in telomere length. In addition, the over-expressed PML C/C^-^ could not perturb the endogenous PML in the U2OS cells to affect the inherent telomere maintenance pathway. This suggests that there is possibly an optimal level of PML in the ALT cells that could sustain the ALT pathway. We also showed that the coiled-coil domain of PML is required for the formation of distinct PML foci and this is consistent with published reports [21,23,24]. When PML does not form distinct nuclear foci, no APBs were observed in our study.

We report for the first time that the over-expression of wild-type PML and PML C/C^-^ in telomerase positive MCF7 cells led to rapid telomere elongation. Wild-type PML formed APBs in MCF7 cells stably expressing the protein and the cells demonstrated a corresponding rapid increase in telomere length. In MCF7 cells, while PML C/C^-^ does not form APBs, the protein can also result in telomere elongation. Our data suggests that the stable expression of wild-type PML could possibly bring about two greatly differing phenotypes in MCF7 cells. Wild-type PML could possibly either cause a heightened activity of the telomerase enzyme or it could form APBs in MCF7 cells and bring about rapid elongation of telomeres. The latter suggestion would add on to the existing knowledge that the occurrence of the ALT pathway is tightly correlated with the presence of APBs [18,28]. However, the stable expression of PML C/C^-^ in MCF7 cells also led to rapid telomere elongation and this happened in the absence of APBs. This observation thus offers the view that APBs might not be absolutely essential for the ALT pathway. Indeed, it has been reported that some cancer cells exhibit telomere maintenance mechanism that is telomerase-independent which occurs in the absence of APBs [15,16]. It is noted that while telomere length increment in PML C/C^-^ STC 22 cells is comparable to that in PML STC10, there is gradual accumulation of longer telomeres in the former. We postulate that while wild-type PML could elicit an all-or-nothing response in MCF7 cells, PML C/C^-^ might possess an unknown quality that allows the cells expressing it to exhibit telomere lengthening while bypassing the requirement for APBs. However, it is possible that PML C/C^-^ might not bring about a rapid increment in telomere length compared to the wild-type protein, and it might require more time for the accumulation of longer telomeres. More work definitely has to be done to determine if similar results could be obtained in other telomerase-positive cell types and to elucidate the molecular players involved in the switch in the telomere maintenance mechanisms. We also acknowledge that our observed phenotypes could be subjected to clonal specificity. It is, however, clear that the over-expression of either wild-type PML or the PML C/C^-^ can definitely affect the telomere maintenance pathway in telomerase-positive MCF7 cells while not significantly disrupting that in ALT-positive U2OS cells.

Finally, our finding that the MCF7 cells with significantly longer telomeres, PML STC10 and PML C/C^-^ STC22, displayed enhanced sensitivity towards doxorubicin implies that certain compounds could exhibit selectivity towards cells with extremely long telomeres. In light of the development of the use of telomerase inhibitors in cancer therapy [29], there is the possibility of the emergence of telomerase inhibitor-resistant cells which could switch to ALT pathway, thereby ending up with unusually long telomeres. More studies are needed to determine whether unusually long telomeres could indeed be a drug target in cancer cells that utilise the ALT pathway and in cells that have demonstrated a possible switch in the telomere maintenance mechanism. In addition, the use of more advanced techniques, such as Q-FISH (quantitative fluorescence in situ hybridisation) and STELA (single telomere length analysis), in such studies would be advantageous in determining the actual changes in telomere length that might have occurred during and after the treatment process.

Our observations suggest that APBs might not be essential for the ALT pathway, although its presence could make the microenvironment favourable for the pathway. In addition, the PML protein could be a very important protein for the ALT pathway and its role might not be confined to that of APBs formation alone. As such, the PML protein could be a new investigational target in the study of the ALT pathway. It would be interesting to study if there is a correlation between PML expression and telomerase levels and activity in cancer tumours. Further work in other telomerase positive cell lines (multiple clones) and tumours will help to clarify of the role of PML in influencing the existing telomerase pathway.

## Methods

### Cell culture, transfection and expression vectors

U2OS, ALT-positive osteosarcoma cells (ATCC) and JFCF-6/T.1R cells (gift from Prof. R. Reddel) [8] were grown in 5A McCoy’s medium, supplemented with 10% foetal bovine serum and L-glutamine. Cells were sub-cultured when 80% confluent with trypsin-EDTA. Cells were maintained in 37°C incubator with 5% atmospheric CO_2_. Cells were seeded at 70–80% confluency for transfections.

Lipofectamine 2000 (Invitrogen) was used for transfection of all cell-lines. Lipofectamine 2000 was used at a 1:3 ratio, with 3 μl used with every 1 μg of plasmid. Briefly, Lipofectamine 2000 reagent was diluted in Opti-MEM (Invitrogen) and added to plasmids to be used for transfection. The mixture was allowed to incubate for 45 min, after which the entire mixture was added to cell cultures. Cells were harvested 24, 48 or 72 h after transfection. The same procedure was followed for stable transfections except that 48 h after transfection, the selection antibiotic, G418, was added for 14–21 days. The mixed population obtained after 14–21 days of selection was then seeded at 1000 cells per 15-cm plate and colonies were allowed to form in the presence of G418. When colonies were visible, they were picked and amplified. Western blot was performed to ensure the stable integration of the plasmid transfected. Cells were maintained in media containing G418.

Wild-type PML (isoform IV) and PML C/C^-^ expression vectors were used in this study. Wild-type PML and PML KR mutants are tagged with haemagglutinin (HA) while the coiled-coil deficient PML is tagged with the FLAG peptide.

### PCR mutagenesis

There are three established lysine sites in PML which are modifiable by SUMO; lysines at position 65, 160 and 490. PCR mutagenesis was performed to generate the single PML KR mutants using the wild-type PML vector. For generation of the double mutants, the plasmids containing single mutants were used. For generation of the triple mutant, the plasmids containing double mutations were used. The mutations to be introduced by the primers are underlined accordingly. For mutating lysine at position 65 to arginine, the sequence of the forward primer used was 5′ CAGGCGGAAGCCCGATGCCCGAAGCTG 3′ and the reverse primer was 5′ CAGCTTCGGGCA TCGGGCTTCCGCCTG 3′. For mutating lysine at position 160, the forward primer used was 5′ CAGTGGTTCCTC CGTCACGAGGCCCGG 3′ and the reverse primer used was 5′ CCGGGCCTCGTG ACGGAGGAACCACTG 3′. For mutating lysine at position 490, the forward primer used was 5′ AGGAAGGTCATC CGCATGGAGTCTGAG 3′ and the reverse primer used was 5′ CTCAGACTCCAT GCGGATGACCTTCCT 3′. PCR was carried out with the wild-type PML plasmid, the forward and reverse primers, dNTPs and Pfu Turbo enzyme (Invitrogen). The PCR conditions are as follow; for initialization, 95°C for 30 s, denaturation at 95°C for 30 s, annealing at 55°C for 1 minute and extension at 68°C for 30 min for 18 cycles. The PCR products were then digested with DpnI to digest the parental wild-type PML plasmid. The plasmids were then sequenced with the ABI PRISM Big Dye Reaction Terminator CycleSequencing Kit version 3.1 (Applied Biosystems) to verify the success of the mutagenesis process.

### Western blotting

Cells were dislodged using trypsin-EDTA and lysed with lysis buffer containing PMSF, DTT and complete-MINI tablet (Roche). Equal amounts of proteins were loaded into 10% SDS-polyacrylamide gel and separated at 220 V for about 50 min. Proteins were then transferred onto a nitrocellulose membrane via semi-dry transfer. Membranes were then placed in blocking solution containing milk and TBS and subsequently incubated with primary antibodies at 4°C overnight. On the second day, the membrane was incubated with secondary antibodies for one hour at room temperature. Proteins were then detected with the use of SuperSignal West Femto substrate (Thermo Scientific). Antibodies used were anti-HA (1:2000; Covance), anti-FLAG (1:6000; Sigma), anti-actin (1:2000; Sigma), anti-SUMO (1:500; Zymed) antibodies.

### Immunofluorescence

Ten thousand cells were seeded onto a coverslip and allowed to attach overnight. After cells were fixed with 4% paraformaldehyde for 10 min at room temperature, they were permeabilised with 0.1% Triton X-100. After 10 min of incubation with 3% BSA, cells were then incubated with primary antibodies for 1 h at room temperature and subsequently, with fluorescent secondary antibodies for another hour at room temperature. After a series of washing, the slides were left to dry. Thereafter, mounting media containing DAPI (Vectashield) was added and coverslips were then mounted onto glass slides and examined under confocal fluorescent microscope. Images were captured at the 100X magnification. Primary antibodies used were anti-PML (1:100; PG-M3, Santa Cruz), anti-TRF2 (1:100; Santa Cruz), anti-HA (1:5000; Covance), anti-FLAG (1:10000; Sigma). Secondary antibodies used were Alexa Fluor 488 (1:1000; Invitrogen) and Alexa Flour 594 (1:1000; Invitrogen).

### Terminal restriction fragment analysis and southern blot

TeloTAGGG Telomere length assay kit (Roche) was used for the measurement of telomere length. Instructions were followed per manufacturer’s kit. Briefly, genomic DNA was digested with HinfI and RsaI for 10–15 min in 37°C water bath. Digested genomic DNA was then separated in 0.7% agarose gel for at 60 V for about six hours. The gel was then placed in an acidic solution and after neutralisation, the gel was placed in a ‘sandwich’ for southern transfer of DNA onto a membrane via capillary action. Following overnight transfer, DNA on membrane was fixed by UV and incubated with DIG labelled anti-TTAGGG probe at 42°C for at least 4 h. Visualisation was done with the anti-DIG chemiluminescent substrate, CDP-STAR. The average telomere length was determined by comparison with the molecular weight standard using Kodak Imager.

### Telomerase activity assay

Telomerase activity was determined with the TRAPeze XL kit from Chemicon. The protocol per manufacturer’s instructions was followed. Briefly, protein lysate was prepared for a PCR reaction with Amplifluor® primers. The thermocycler was set for a 4-step PCR as follows; 94°C/30 s, 59°C/30 s, 72°C/1 min for 36 cycles followed by a 72°C/3 min extension step and then 55°C/25 min, concluding with a 4°C incubation. Following the PCR reaction, equal amounts of the reaction mix consisting of 10 mM Tris–HCl pH 7.4, 0.15 M NaCl and 2 mM MgCl_2_ was added to the PCR reaction mixture. The fluorescent readout was obtained with Magellan, with excitation/emission parameters for fluorescein set at 495 nm/516 nm and sulforhodamine set at 600 nm/620 nm. Analysis was performed following manufacturer’s instructions.

### Crystal violet cell viability assay

Crystal violet intercalates into double-stranded intact DNA and was used to assess the viability of the cells after treatment with doxorubicin and tamoxifen. Cells were seeded at 0.1 × 10^6^ cells in 6-wells plate and allowed to attach overnight. Cells were then treated with either doxorubicin or tamoxifen for 24 and 48 h. After the desired treatment duration, media was removed and cells were washed with PBS twice. 500 μl of crystal violet solution was added to the wells and incubated at room temperature for 15 min. After a series of washes, the cells were then dissolved in 0.1% triton-× 100 (diluted with PBS) and the absorbance was measured at 595 nm.

### Statistical significance

Student’s *t*-test (Graphpad Prism) was used to assess statistical significance.

## Competing interests

Prakash Hande is one of the Editors-in-Chief of Genome Integrity. This manuscript is processed and handled by the Co-Editor-in-Chief who made the final decision on the acceptance. The author had no input in the process or decision. The authors declare that they have no other competing interests.

## Authors’ contributions

JWYY, MBL and MPH designed the experiments. JWYY and XY performed the experiments. MMK provided plasmids and together with MPH analysed the data. JWYY wrote the manuscript and MPH and MMK edited it. All the authors approved the final version of the manuscript.
